# Zulu ocular biometry differs fundamentally from that of Europeans – A modelling analysis

**DOI:** 10.1111/opo.70000

**Published:** 2025-08-08

**Authors:** Veronica Lockett-Ruiz, Tanya Evans, Rafael Navarro, Khathutshelo Percy Mashige, Jos J. Rozema

**Affiliations:** 1 INMA-CSIC and Universidad de Zaragoza, Zaragoza, Spain; 2 Department of Optometry, University of Johannesburg, Johannesburg, South Africa; 3 School of Health Sciences, University of KwaZulu-Natal, Durban, South Africa; 4 Visual Optics Lab Antwerp (VOLANTIS), Faculty of Medicine and Health Sciences, University of Antwerp, Wilrijk, Belgium; 5 Department of Ophthalmology, Antwerp University Hospital, Edegem, Belgium; 6 Institute for Medical Informatics, Statistics and Epidemiology (IMISE), Leipzig University, Leipzig, Germany; 7 University of Antwerp, Wilrijk, Belgium

**Keywords:** African, axial length, corneal thickness, eye, ocular biometry, refractive error

## Abstract

**Purpose:**

The accuracy of ocular refraction calculations depends on reliable biometry and the validity of the models upon which the calculations are based. Most models are based on European eyes, so their validity in sub-Saharan Africans may be questioned, potentially contributing to suboptimal postoperative outcomes. Hence, this study developed an optical eye model tailored to a Zulu cohort.

**Methods:**

Ocular biometry data were collected from 192 near-emmetropic (i.e., non-cycloplegic spherical equivalent within ±1D) young individuals of Zulu descent in Durban, South Africa. Key biometric parameters, including axial length, corneal thickness, anterior chamber depth, crystalline lens thickness and vitreous chamber depth, were measured and input into Bennett's and Royston's equations to develop the eye model. This model was subsequently compared to the eyes of 32 near-emmetropic young European adults that were used previously to help develop the SyntEyes model. Statistical analysis was performed to identify significant differences in ocular structural parameters between the models.

**Results:**

Zulu eyes exhibited marked differences compared to European eyes. On average, Zulu eyes had deeper anterior chambers (+0.28 mm, *t*-test, *p* < 0.001), thinner crystalline lenses (−0.19 mm, *p* < 0.001) and lower corneal power (−0.83 D, *p* = 0.001). However, the total ocular power of Zulu eyes was greater due to a more powerful crystalline lens (+2.60 D, *p* < 0.001). No significant difference in refractive error or axial length was observed between the two populations.

**Conclusions:**

The ocular biometry of the Zulu population differs significantly from that of the European population, particularly in corneal and crystalline lens power, lens thickness and anterior chamber depth. This has important implications for intraocular lens power calculations, glaucoma risk assessment and other diagnostic procedures. Accurate, population-specific references are essential for optimising vision correction and enhancing ophthalmic healthcare outcomes in regions with populations underrepresented in the literature.

## Key points

- To date, little information is available in the literature regarding ocular biometry in sub-Saharan Africans.
- This work presents the first refractive eye model for Africans based on previous biometry measurements in a Zulu cohort.
- The power balance in Zulu eyes is significantly different from that in European eyes, with relatively less power in the cornea and more in the crystalline lens.

## INTRODUCTION

The availability of accurate and precise ocular biometry is crucial for effective eye treatment and management of ocular diseases. Several eye care procedures, such as intraocular lens (IOL) power calculations, myopia management and interpretation of intraocular pressure (IOP) measurements, depend on accurate ocular biometry. Biometry may differ between ethnic groups, as over the past few decades, studies from North America and Europe have consistently reported that individuals of African descent have thinner corneas than those of European ancestry.^1–8^ Central corneal thickness (CCT) influences IOP measurements, with thinner corneas yielding lower IOP readings.^9,10^ This discrepancy may contribute to an underestimation of IOP, delayed glaucoma diagnosis and poor glaucoma prevention in populations with typically thinner corneas.^8^ Beyond corneal thickness, studies conducted in the United States,^11^ Nigeria^12,13^ and South Africa^7,14^ have also reported that individuals of African heritage have a shorter ocular axial length (AL) for the same refractive error.

A Delphi study conducted amongst clinicians in sub-Saharan Africa highlighted ocular biometry as a key factor in enhancing cataract surgery quality and patient outcomes within the region.^15^ While ocular biometry is widely integrated into clinical practice in most parts of the world, its systematic implementation remains limited in sub-Saharan Africa.^15^ Consequently, clinicians in the region rely on biometric reference values derived from European, Asian and American populations, which may not be representative of local ocular characteristics. This reliance could result in poor postoperative outcomes in cataract surgery patients.^7^ The availability of region-specific ocular biometric data may enhance the accuracy of preoperative assessments, improve preventive measures for ocular diseases, and help refine clinical risk stratification in glaucoma management, leading to better patient outcomes.^8^

To achieve this objective, a biometric eye model was developed based on the measurements of healthy young adult individuals of Zulu descent. This research is essential for understanding the differences in ocular structure and function across diverse populations and how these differences can impact the diagnosis and management of ocular diseases.

## MATERIALS AND METHODS

### Participants and measurements

The data used for this study were collected from a prior biometric survey^7^ conducted in Durban, a major city in the KwaZulu-Natal province of South Africa. The study was conducted at the eye clinic operated by the Discipline of Optometry at the Westville Campus of the University of KwaZulu-Natal (UKZN). The study population was comprised of Black South African participants of Zulu ethnicity, recruited from geographically contiguous areas of Durban, encompassing urban, peri-urban and rural settings. The Zulu people, an indigenous Nguni-speaking population of Southern Africa, represent the largest ethnic group in South Africa, predominantly residing in the KwaZulu-Natal province. The study included young adults of Zulu ancestry with healthy eyes, IOP < 21 mmHg and no history of ocular surgery or disease. This survey was approved by the University of KwaZulu-Natal's Biomedical and Research Ethics Committee (ref: BEE311/312). All participants gave informed consent prior to their inclusion.

The data analysed to create the model consisted of 192 right eyes from individuals of Zulu descent, with women accounting for 47.9%. Participants were selected through a randomised, stratified, cluster sampling process from six districts of Durban, KwaZulu-Natal Province, South Africa. The inclusion criteria for this analysis were limited to individuals with near-emmetropic and emmetropic eyes (i.e., spherical equivalent refractive error between ±1 D), aged between 20 and 25 years. All participants underwent ocular biometry measurements, which included parameters such as the spherical equivalent (SE) refractive error, anterior corneal radius (*r*_ca_), CCT, anterior chamber depth (ACD), near-emmetropic lens thickness (LT) and AL. The non-cycloplegic SE refractive error was measured using an autorefractometer (Nidek AR-310A; nidek-intl.com) and the anterior corneal radius was estimated using a keratometer (Oculus Keratograph 4; oculus.de). CCT was measured using optical coherence tomography (OCT) (Optovue iVue-100; visionix.com), while ultrasound (Nidek US-500) was used to measure ACD, LT and AL.

For comparative analysis, the raw biometric data of Belgian eyes was used to create the SyntEyes eye model.^16^ This is presented alongside the Zulu dataset to elucidate differences in ocular biometry between the two populations. As SyntEyes is based on adult data of individuals aged between 20 and 30 years with non-cycloplegic refractive errors between ±10D, a selection from this cohort was used, consisting of near-emmetropic eyes (i.e., SE between ±1D) from individuals aged between 20 and 30 years. This resulted in 32 right eyes of 15 males and 17 female participants, who had their biometry measured with the Pentacam HR (Oculus) and the Lenstar (LS-900, haag-streit.com), while their refractive error was measured using an autorefractometer (Nidek AR-700).

Only the non-cycloplegic SE was available for both cohorts and given that an average myopic shift of 0.40 D is expected to occur in non-cycloplegic SE relative to the cyclopleged SE,^17^ a value of +0.40 D was added to the refractive error data to simulate the loss of accommodative tonus from cycloplegia.

### Modelling

Model development was based on the methods of Bennett^18,19^ and Royston,^20^ using the refractive index values and proportions taken from the SyntEyes model.^16^ Using the notations detailed in Table 1, with length in millimetres and optical power in dioptres, the total corneal power can be estimated as:

1 $$ {P}_{\mathrm{c}}=1000\cdotp \left({n}_{\mathrm{k}}-1\right)/{r}_{\mathrm{c}\mathrm{a}} $$

with *n*_k_ = 1.3294, the keratometric index value, derived from the Pentacam, to compute the total corneal power for the Zulu data sets. This value is similar to that proposed by Dubbelman et al.^21^ (*n*_k_ = 1.329). The anterior corneal power is given by:

2 $$ {P}_{\mathrm{c}\mathrm{a}}=1000\cdotp \left({n}_{\mathrm{c}}-1\right)/{r}_{\mathrm{c}\mathrm{a}} $$

where *n*_c_ = 1.376 is the refractive index of the cornea. Rearranging the thick lens equation, the posterior corneal power can then be estimated as:

3 $$ {P}_{\mathrm{c}\mathrm{p}}=\frac{P_{\mathrm{c}}-{P}_{\mathrm{c}\mathrm{a}}}{1-0.001\cdotp {P}_{\mathrm{c}\mathrm{a}}\mathrm{CCT}/{n}_{\mathrm{c}}} $$

from which the posterior corneal radius of curvature can be computed as:

4 $$ {r}_{\mathrm{c}\mathrm{p}}=1000\cdotp \left({n}_{\mathrm{a}}-{n}_{\mathrm{c}}\right)/{P}_{\mathrm{c}\mathrm{p}} $$

with *n* = 1.336, the refractive index of the ocular humours. This completes the corneal parameters.

**TABLE 1:** Model parameters assuming the refractive indices of the SyntEyes model.^16^

Symbol	Unit	Data source	Zulu			Europeans (all)	Description
					Men	Women	All
*N*	–	–	100	92	192	32	Number of near-emmetropic eyes used
Age	years	–	20.8	20.6	20.7	25.8	Average age of participants
*r*_ca_	mm	Meas.	7.99	7.81	7.91	7.74	Anterior corneal radius of curvature
*r*_cp_	mm	Calc.	6.81	6.66	6.74	6.55	Posterior corneal radius of curvature
*r*_la_	mm	Calc.	9.76	9.41	9.59	10.67	Anterior lenticular radius of curvature
*r*_lp_	mm	Calc.	−6.41	−6.19	−6.30	−7.02	Posterior lenticular radius of curvature
CCT	mm	Meas.	0.488	0.485	0.487	0.539	Central corneal thickness
ACD	mm	Meas.	3.42	3.30	3.36	3.08	Anterior chamber depth (excl. cornea)
LT	mm	Meas.	3.53	3.51	3.52	3.71	Lenticular thickness
VCD	mm	Meas.	16.17	15.67	15.92	16.23	Vitreous chamber depth
RET	mm	Model	0.20	0.20	0.20	0.20	Retinal thickness
AL	mm	Meas.	23.81	23.17	23.50	23.77	Axial length (up to photoreceptor layer)
*n*_c_	–	Model	1.376	1.376	1.376	1.376	Refractive index of the cornea
*n*_k_	–	Model	1.3294	1.3294	1.3294	1.3294	Keratometric index
*n*_a_	–	Model	1.336	1.336	1.336	1.336	Refractive index of aqueous and vitreous
*n*_l_	–	Model	1.4335	1.4335	1.4335	1.4335	Equivalent refractive index of the lens
*P*_ca_	D	Calc.	47.04	48.14	47.56	48.55	Anterior cornea surface power
*P*_cp_	D	Calc.	−5.87	−6.01	−5.93	−6.11	Posterior cornea surface power
*P*_c_	D	Calc.	41.26	42.23	41.73	42.56	Total corneal power
*pp*_c1_^a^	mm	Calc.	−0.05	−0.05	−0.05	−0.06	1st corneal principal point position
*pp*_c2_^a^	mm	Calc.	−0.05	−0.05	−0.05	−0.06	2nd corneal principal point position
*P*_la_	D	Calc.	9.99	10.37	10.17	9.13	Anterior lenticular surface power
*P*_lp_	D	Calc.	15.20	15.79	15.47	13.90	Posterior lenticular surface power
*P*_l_	D	Calc.	24.82	25.76	25.25	22.70	Total crystalline lens power
*pp*_l1_^a^	mm	Calc.	5.93	5.79	5.86	5.74	1st lenticular principal point position
*pp*_l2_^a^	mm	Calc.	6.12	5.98	6.05	5.94	2nd lenticular principal point position
Δ	mm	Calc.	0.020	0.010	0.020	0.017	Offset parameter
*P*_eye_	D	Calc.	61.50	63.23	62.32	60.07	Whole eye power
*pp*_eye1_^a^	mm	Calc.	1.76	1.73	1.74	1.56	1st ocular principal point position
*pp*_eye2_^a^	mm	Calc.	2.11	2.08	2.09	1.90	2nd ocular principal point position
*P*_ax_	D	Calc.	61.55	63.34	62.39	61.11	Axial power (Dioptric distance)
SE	D	Meas.	0.05	0.11	0.08	0.04	Spherical equivalent refractive error
*f*_eye1_	mm	Calc.	−14.50	−14.08	−14.30	−14.82	1st ocular focal point position
*f*_eye2_	mm	Calc.	23.83	23.20	23.53	23.78	2nd ocular focal point position
*n*_eye1_^a^	mm	Calc.	7.22	7.04	7.14	7.06	1st ocular nodal point position
*n*_eye2_^a^	mm	Calc.	7.57	7.39	7.48	7.40	2nd ocular nodal point position
*p*_entpup_^a^	mm	Calc.	3.33	3.22	3.28	3.06	Entrance pupil position
*p*_extpup_^a^	mm	Calc.	4.02	3.89	3.96	3.75	Exit pupil position
*D*_entpup_	–	Calc.	1.137×	1.136×	1.137×	1.130×	Entrance pupil size
*D*_extpup_	–	Calc.	1.039×	1.040×	1.039×	1.037×	Exit pupil size

^a^Distance considered from the anterior corneal apex; Meas.: measured data; Calc. calculated data.

Next, the crystalline lens power can be computed using Bennett's equation^18,19^ by adding the retinal thickness (RET) to the vitreous chamber depth (VCD), as the axial length is typically reported up to the internal limiting membrane rather than the photoreceptor plane. The modified equation for the crystalline lens power is as follows:

5 $$ {\displaystyle \begin{array}{c}{P}_{\mathrm{L}}=\frac{-1000\cdotp n\ \left({S}_{\mathrm{CV}}+{P}_{\mathrm{c}}\right)}{1000\cdotp n-\left(\mathrm{ACD}+{c}_1\cdotp \mathrm{LT}\right)\ \left({S}_{\mathrm{CV}}+{P}_{\mathrm{c}}\right)}\hfill \\ {}\kern2em +\frac{1000\cdotp n}{-{c}_2\cdotp \mathrm{LT}+\mathrm{VCD}+\mathrm{RET}}\hfill \end{array}} $$

where *c*_1_ = 0.571 and *c*_2_ = −0.375 are the relative positions of the lenticular principal planes within the crystalline lens based on the SyntEyes model. *S*_CV_ is the spherical refraction at the corneal vertex, given by:

6 $$ {S}_{\mathrm{CV}}=\mathrm{SE}/\left(1-0.014\cdotp \mathrm{SE}\right) $$

assuming a vertex distance of 14 mm. From equation (5), the lens radii of curvature can then be calculated using Royston's equations,^20^ again using constants *c*_1_ and *c*_2_:

7 $$ \left\{\begin{array}{c}{r}_{\mathrm{l}\mathrm{a}}=\frac{1000\cdotp \left({n}_{\mathrm{l}}-n\right)}{Q{P}_{\mathrm{l}}}+\Delta  \\ {}{r}_{\mathrm{l}\mathrm{p}}=\frac{1000\cdotp {c}_2\left({n}_{\mathrm{l}}-n\right)}{c_1Q{P}_{\mathrm{l}}}-\Delta  \end{array}\right. $$

where *Q* = 0.402 is the anterior lens surface power ratio to the total crystalline lens power and *n*_l_ = 1.4335 is the lens' equivalent refractive index. Finally, Δ is an offset parameter required to fine-tune the model to an overall refractive error that matches the average value measured in the population.

### Statistics

All comparisons between the two datasets were conducted using *t*-tests in SPSS (v25, ibm.com), with a significance threshold of 0.05/8 = 0.006 (Bonferroni correction). Due to the considerable difference in cohort size of 192 Zulu vs. 32 Europeans, the variance of both cohorts was found to be unequal (Levene test, *p* < 0.001). Hence, Welch's *t*-test was used instead of Student's *t*-test as the former does not require equal variances.

## RESULTS

**FIGURE 1:**
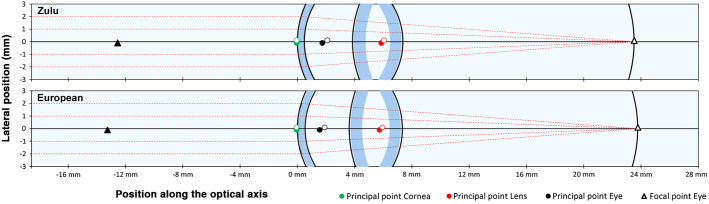
Overview of the Zulu eye model in comparison with the SyntEyes (European) model. Solid markers represent the first cardinal points, while open markers represent the second cardinal points.

An overview of the model parameters and the cardinal points for men, women and both sexes is shown in Table 1, along with the European data. While macroscopic differences in ocular biometry between the Zulu and European cohorts appear minimal (Figure 1), many parameters exhibit statistically significant differences (Table 2). On average, Zulu eyes had significantly deeper anterior chambers than European eyes by 0.28 mm (unpaired *t*-test, 0.28 mm, *p* < 0.001) and thinner crystalline lenses (−0.19 mm, *p* < 0.001). However, no significant differences were observed for SE (*p* = 0.34), AL (*p* = 0.04) or VCD (*p* = 0.01). For the same AL and SE, the crystalline lens exhibited a significantly higher power in the Zulu cohort (+2.60 D, *p* < 0.001), whereas the corneal power was unexpectedly lower (−0.83 D, *p* < 0.001). Additionally, the well-documented difference in CCT^1–8^ was observed (−0.052 mm, *p* < 0.001), but this holds minimal optical significance.

**TABLE 2:** Comparison of the raw data for the Zulu and European cohorts (mean ± standard error).

Parameter	Unit	Zulu data	European data	Difference	*t*-test^a^
SE refractive error	D	0.08 ± 0.42	0.04 ± 0.37	0.04	0.58
Central corneal thickness	mm	0.487 ± 0.032	0.539 ± 0.034	−0.052	**<0.001**
Anterior chamber depth	mm	3.36 ± 0.21	3.08 ± 0.32	0.28	**<0.001**
Crystalline lens thickness	mm	3.52 ± 0.20	3.71 ± 0.19	−0.19	**<0.001**
Vitreous chamber depth	mm	15.93 ± 0.76	16.23 ± 0.59	−0.30	0.01
Axial length^b^	mm	23.50 ± 0.79	23.77 ± 0.64	−0.26	0.04
Total corneal power	D	41.73 ± 1.61	42.56 ± 1.06	−0.83	**<0.001**
Total crystalline lens power	D	25.35 ± 2.05	22.76 ± 1.99	2.60	**<0.001**

^a^Unpaired *t*-test assuming unequal variances, where *p* < 0.05/8 = 0.006 is considered significant (Bonferroni correction; in boldface).

^b^Including 0.2 mm of retinal thickness. D, dioptres; SE, spherical equivalent refractive error.

## DISCUSSION

To the best of our knowledge, the model presented in this paper is the first of its kind based on the Zulu population or any other population in sub-Saharan Africa. Overall, Zulu eyes exhibit a greater optical power, characterised by thinner crystalline lenses that contribute more to the total ocular refraction compared with their European counterparts (40.5% vs. 37.1% contribution to the total eye power, respectively), while the corneal power is lower in Zulu eyes due to a larger anterior corneal radius of curvature. Despite the shorter axial length observed in Zulu eyes, their anterior chamber depth was unexpectedly deeper compared to European eyes. These differences are clinically highly significant and may lead to very poor clinical outcomes if disregarded.

These observations suggest that there may be important differences in the internal dioptric structures of the eyes between the studied populations, which may affect the accuracy of refractive power calculations and impact postoperative outcomes in Zulu eyes.^22–24^ This could be addressed by optimising the calculation constants used and re-evaluating formula selection criteria to consider the unique proportions of the Zulu eyes. Furthermore, these structural differences should also be included in the calibration of diagnostic instruments to screen for glaucoma,^25^ monitor myopia control and assess for keratoconus in this demographic.

While sub-Saharan African populations exhibit considerable genetic and biometric diversity,^26^ similar concerns may be relevant to other populations within the region. Recently, Asano et al.^27^ reported biometric data for 657 eyes from individuals living in the rural area of Mkuranga, Tanzania, with a mean age of 57.2 years. While refractive error and crystalline lens thickness values were not provided, the lens power can be estimated using the Bennett-Rabbetts equation,^18^ assuming emmetropia and the same keratometric index presented in Table 1. This calculation yields an estimated power of 23.99D, notably higher than the values found in Western literature and the European cohort (22.70D). Moreover, considering the nearly 30-year age difference between the Zulu and Asano et al. datasets, it is reasonable to speculate that these Tanzanian eyes may also have had crystalline lens powers of about 25D at age 20 years. However, as these populations live approximately 2500 km apart, they may have considerable genetic differences, potentially making it inappropriate to draw such parallels.

This study has several limitations. First, using non-cycloplegic biometric data may have influenced the computed refractive error,^28^ as well as the ACD and LT, due to residual accommodation that differs between individuals. To mitigate this issue, a mean estimated offset of +0.40D was applied to the refractive error of both cohorts. Note, however, that this is a mean estimate and that there may be considerable differences in individual eyes. The overall errors due to ACD and LT for minor degrees of accommodation were likely minor (about 0.02–0.05 mm)^29^ and thus could be disregarded. Second, there is a 5-year age disparity between the Zulu (mean age: 20.7 years) and the European participants (mean age: 25.8 years), which may account for some of the variations observed in Table 2, but only to a minor degree. Third, the large size difference between the cohorts could reduce the statistical power and effect size of the study. While this may perhaps have underpowered the comparisons of AL and VCD, the key differences reported in Table 2 for the positions and refractive powers of the cornea and crystalline lens were highly significant and therefore not affected by cohort size. Fourth, the crystalline lens power calculations assume a fixed anterior-to-posterior lens radius ratio, as in the European eyes taken from the SyntEyes model. While this ratio may vary somewhat between populations around the world, it is unlikely to lead to large errors. This is supported by evidence that the Bennett equation^18,19^ remains accurate despite age-related^30^ or refractive error-related^31^ changes in adult eyes, which are likely larger than the average differences between populations. Finally, the ocular biometry in the Zulu cohort was measured using ultrasound, while the European data were determined by optical biometry. While ultrasound has a poorer repeatability than optical methods and may affect individual measurements, the overall average of both methods should be comparable.

Note also that the higher crystalline lens power in Zulu eyes may either result from steeper surface curvatures or from a higher equivalent refractive index, but the current data cannot be used to determine the most likely explanation. Should the latter hypothesis be true, then the radii of curvature of the Zulu eye model will have to be flatter than the values given in Table 1, as the Bennett-Royston^18–20^ modelling scheme assumes a fixed value for the equivalent refractive index.

This study delineates distinct ocular characteristics in the Zulu population critical for accurate diagnosis and effective treatment. The modelling approach used here can be applied to biometric data from other adult populations, supporting the development of population-specific ocular models and advancing eye care in underrepresented regions. Future work should aim to incorporate cycloplegic data, age-matched comparisons and direct lens measurements to improve model accuracy and clinical applicability further.

## References

[1] La Rosa FA, Gross RL, Orengo-Nania S. Central corneal thickness of Caucasians and African Americans in glaucomatous and nonglaucomatous populations. *Arch Ophthalmol.* 2001;119:23–27. 11146722

[2] Shimmyo M, Ross AJ, Moy A, Mostafavi R. Intraocular pressure, Goldmann applanation tension, corneal thickness and corneal curvature in Caucasians, Asians, Hispanics and African Americans. *Am J Ophthalmol.* 2003;136:603–613. 14516799 10.1016/s0002-9394(03)00424-0

[3] Aghaian E, Choe JE, Lin S, Stamper RL. Central corneal thickness of Caucasians, Chinese, Hispanics, Filipinos, African Americans and Japanese in a glaucoma clinic. *Ophthalmology.* 2004;111:2211–2219. 15582076 10.1016/j.ophtha.2004.06.013

[4] Mercieca K, Odogu V, Fiebai B, Arowolo O, Chukwuka F. Comparing central corneal thickness in a sub-Saharan cohort to African Americans and Afro-Caribbeans. *Cornea.* 2007;26:557–560. 17525651 10.1097/ICO.0b013e3180415d90

[5] Sample PA. The African Descent and Glaucoma Evaluation Study (ADAGES): design and baseline data. *Arch Ophthalmol*. 2009;127:1136–1145. 19752422 10.1001/archophthalmol.2009.187 PMC2761830

[6] Iyamu E, Osuobeni E. Age, gender, corneal diameter, corneal curvature and central corneal thickness in Nigerians with normal intra ocular pressure. *J Optom.* 2012;5:87–97.

[7] Mashige KP, Oduntan OA. Axial length, anterior chamber depth and lens thickness: their intercorrelations in black South Africans. *Afr Vis Eye Health.* 2017;76:a362. 10.4102/aveh.v76i1.362

[8] Baboolal SO, Smit DP. South African eye study (SAES): ethnic differences in central corneal thickness and intraocular pressure. *Eye*. 2018;32:749–756. 29328064 10.1038/eye.2017.291 PMC5898866

[9] Wolfs RCW, Klaver CCW, Vingerling JR, Grobbee DE, Hofman A, de Jong PTVM. Distribution of central corneal thickness and its association with intraocular pressure: the Rotterdam study. *Am J Ophthalmol.* 1997;123:767–772. 9535620 10.1016/s0002-9394(14)71125-0

[10] Doughty MJ, Zaman ML. Human corneal thickness and its impact on intraocular pressure measures: a review and meta-analysis approach. *Surv Ophthalmol.* 2000;44:367–408. 10734239 10.1016/s0039-6257(00)00110-7

[11] Oliveira C, Harizman N, Girkin CA, Xie A, Tello C, Liebmann JM, et al. Axial length and optic disc size in normal eyes. *Br J Ophthalmol.* 2007;91:37–39. 16987902 10.1136/bjo.2006.102061 PMC1857588

[12] Adio AO, Onua DO, Arowolo D. Ocular axial length and keratometry readings of normal eyes in southern Nigeria. *Niger J Ophthalmol.* 2010;18:12–24.

[13] Aiyekomogbon J, Rafindadi A. Ocular axial length measurement among normal adults using magnetic resonance imaging. *Niger J Ophthalmol.* 2017;25:6–10.

[14] Church B, Ballim S, Visser L. Ethnic variation in corneal curvature in Durban. *KwaZulu-Natal South Afr Ophthalmol J.* 2020;15:23–29.

[15] Buchan JC, Dean WH, Foster A, Burton MJ. What are the priorities for improving cataract surgical outcomes in Africa? Results of a Delphi exercise. *Int Ophthalmol.* 2018;38:1409–1414. 28634930 10.1007/s10792-017-0599-y PMC6061020

[16] Rozema JJ, Rodriguez P, Navarro R, Tassignon MJ. SyntEyes: a higher-order statistical eye model for healthy eyes. *Invest Opthalmology Vis Sci.* 2016;57:683–691. 10.1167/iovs.15-18067 26903227

[17] Rozema JJ, Iribarren R, Hashemi H, Khabazkhoob M, Fotouhi A. Mean cycloplegic refractive error in emmetropic adults – the Tehran eye study. *J Optom.* 2024;17:100512. 10.1016/j.optom.2023.100512 38244522 10.1016/j.optom.2023.100512 PMC10832267

[18] Rozema JJ, Atchison DA, Kasthurirangan S, Pope JM, Tassignon MJ. Methods to estimate the size and shape of the unaccommodated crystalline lens in vivo. *Invest Opthalmology Vis Sci.* 2012;53:2533–2540. 10.1167/iovs.11-8645 22427565

[19] Bennett AG. A method of determining the equivalent powers of the eye and its crystalline lens without resort to phakometry. *Ophthalmic Physiol Opt.* 1988;8:53–59. 3047630 10.1016/0275-5408(88)90089-0

[20] Royston JM, Dunne MCM, Barnes DA. Calculation of crystalline lens radii without resort to phakometry. *Ophthalmic Physiol Opt.* 1989;9:412–414. 2631008

[21] Dubbelman M, Sicam VADP, Van der Heijde GL. The shape of the anterior and posterior surface of the aging human cornea. *Vis Res.* 2006;46:993–1001. 16266736 10.1016/j.visres.2005.09.021

[22] Mitra A, Jain E, Sen A, Tripathi S. A study regarding efficacy of various intraocular lens power calculation formulas in a subset of Indian myopic population. *Indian J Ophthalmol.* 2014;62:826–828. 25116783 10.4103/0301-4738.138634 PMC4152660

[23] Wang D, Amoozgar B, Porco T, Wang Z, Lin SC. Ethnic differences in lens parameters measured by ocular biometry in a cataract surgery population. *PLoS One.* 2017;12:e0179836. 10.1371/journal.pone.0179836 28654694 10.1371/journal.pone.0179836 PMC5487046

[24] Kim J, Eom Y, Yoon E, Choi Y, Song JS, Jeong J, et al. Algorithmic intraocular lens power calculation formula selection by keratometry, anterior chamber depth and axial length. *Acta Ophthalmol.* 2021;100:e701–e709. 34378871 10.1111/aos.14956 PMC9292369

[25] Ahmed S, Khan Z, Si F, Mao A, Pan I, Yazdi F, et al. Summary of glaucoma diagnostic testing accuracy: an evidence-based meta-analysis. *J Clin Med Res.* 2016;8:641–649. 27540437 10.14740/jocmr2643w PMC4974833

[26] Campbell MC, Tishkoff SA. African genetic diversity: implications for human demographic history, modern human origins and complex disease mapping. *Annu Rev Genomics Hum Genet.* 2008;9:403–433. 18593304 10.1146/annurev.genom.9.081307.164258 PMC2953791

[27] Asano H, Hiraoka T, Seki Y, Shibata T, Osada H, Saruta T, et al. Distribution of corneal spherical aberration in a Tanzanian population. *PLoS One.* 2019;14:e0222297. 10.1371/journal.pone.0222297 31513608 10.1371/journal.pone.0222297 PMC6742233

[28] Morgan IG, Iribarren R, Fotouhi A, Grzybowski A. Cycloplegic refraction is the gold standard for epidemiological studies. *Acta Ophthalmol.* 2015;93:581–585. 25597549 10.1111/aos.12642

[29] Dubbelman M, Van der Heijde GL, Weeber HA. Change in shape of the aging human crystalline lens with accommodation. *Vis Res.* 2005;45:117–132. 15571742 10.1016/j.visres.2004.07.032

[30] Rozema JJ, Atchison DA. The influence of age on crystalline lens power calculations. *Invest Ophthalmol Vis Sci.* 2016;57:5435. 10.1167/iovs.16-20444 27756078 10.1167/iovs.16-20444

[31] Dunne MCM, Barnes DA, Royston JM. An evaluation of Bennett's method for determining the equivalent powers of the eye and its crystalline lens without resort to phakometry. *Ophthalmic Physiol Opt.* 1989;9:69–71. 2594382 10.1111/j.1475-1313.1989.tb00809.x

